# From birth to triumph: A rare case report of rib chondrosarcoma with unprecedented growth patterns

**DOI:** 10.1097/MD.0000000000042817

**Published:** 2025-06-13

**Authors:** Mei Shen, Ying Liu, Wei Wen, Hanlong Guo, Qu-Cheng Huang, Wen-Juan Liao

**Affiliations:** a Department of Radiography, The Affiliated Taizhou Second People’s Hospital of Yangzhou University, Taizhou, China; b Department of Thoracic Cardiovascular Surgery, General Hospital of Central Theater Command of the People’s Liberation Army, Wuhan, China; c Department of Thoracic Surgery, The First Affiliated Hospital with Nanjing Medical University, Nanjing, China; d Department of the First Clinical Medical College, Gannan Medical University, Ganzhou, China; e Department of Child, The First Affiliated Hospital of Gannan Medical University, Ganzhou, China.

**Keywords:** case report, chondrosarcoma, inert tumor, rib tumors, tumor growth pattern

## Abstract

**Rationale:**

Primary tumors of ribs are uncommon in clinical practice. These tumors can be benign or malignant and often present unique challenges in diagnosis and treatment. For instance, rib chondrosarcoma is a rare type of chondrosarcoma that occurs in the rib cage, representing a significant clinical diagnosis challenge due to its potential for local recurrence and metastasis. With rib chondrosarcoma being more or less common compared to other locations such as the long bones and pelvis. Generally, rib chondrosarcoma develops rapidly and mostly occurred in middle-aged or elderly crowds. Rib chondrosarcoma in children is a rare but significant clinical concern.

**Patient concerns:**

A 30-year-old male patient, prior to marriage, sought surgical removal of a chest wall mass, which present from birth, to achieve an improved aesthetic appearance of the chest wall.

**Diagnoses:**

Preoperative chest computed tomography scans indicated the presence of a rib tumor, which was initially presumed to be benign. However, postoperative histopathological analysis revealed the mass to be a rib chondroma.

**Interventions:**

He received resection of the tumor and reconstruction of chest wall. Considering the slow growth of the tumor, this patient did not receive any other adjuvant treatments after the surgery, including chemotherapy or targeted therapy.

**Outcomes:**

The symptoms disappeared after the operation, no other discomfort was appealed during the follow-up over the next 5 years, and no recurrence of the intrathoracic lesion was detected in the imaging examinations.

**Lessons:**

Such a slow growth pattern of rib chondrosarcoma has not been reported in the previous literature. Considering the diagnosis of rib chondrosarcoma is crucial for assessing the extent of the tumor and planning surgical intervention, this case expands the knowledge of clinicians and radiologists in the diagnosis of rib chondrosarcoma.

## 1. Introduction

The chest wall is rarely affected by malignant primary bone tumors, and rib chondrosarcoma is even rarer. Chondrosarcoma, which is a cartilage matrix-producing tumor, accounts for approximately 15% of all chest wall malignancies.^[1]^ Among these, rib chondrosarcomas can present unique challenges in diagnosis and treatment due to their aggressive nature and the potential for local recurrence.

In children, the presentation of rib chondrosarcoma can be atypical, often resembling benign conditions, which can lead to delays in diagnosis. For instance, imaging techniques such as magnetic resonance imaging (MRI) have proven useful in identifying intramedullary lesions that may not be visible on computed tomography (CT) scans.^[2]^ Moreover, the imaging characteristics of malignant primary chest wall neoplasms, including chondrosarcoma, can be nonspecific, which complicates the diagnostic process.^[2]^ In some cases, such as with giant cell tumors of the rib, the initial presentation may mimic more common conditions, leading to delays in appropriate management.^[3]^

Chondrosarcomas arise from cartilage-producing mesenchymal cells. The incidence rates of chondrosarcoma were 2 parts per million.^[4]^ Generally, the aged at 30 to 60 was high incidence population, and there was no difference in gender.^[4]^ In general, they commonly appear in long bones or the pelvis. Chondrosarcoma of the rib is rare.^[5]^ Furthermore, most studies have included patients with short follow-up, despite the fact that a high rate of late recurrence and metastasis has been reported for chondrosarcoma patients compared to those with other primary bone sarcomas.^[6]^

In this case report, we present a patient of chondrosarcoma of the rib with a mass that found at birth and slowly grew over 30 years. This highlights the importance of utilizing advanced imaging modalities to ensure accurate diagnosis and effective surgical planning.

## 2. Case report

A 30-year-old male patient was hospitalized with a huge mass on the left chest wall. When the patient was born, a mass about the size of a quail egg was found on the left chest wall, and the mass gradually grew up over the past 30 years. Since the patient never presented clinical symptoms and the mass was usually covered by clothes, which did not seriously impact on the patient’s life, so the patient did not treat it during the 30 years. In the past 6 months, the patient gradually developed pain and numbness in the area of the mass, which prompted the patient to come to hospital.

**Figure 1.**
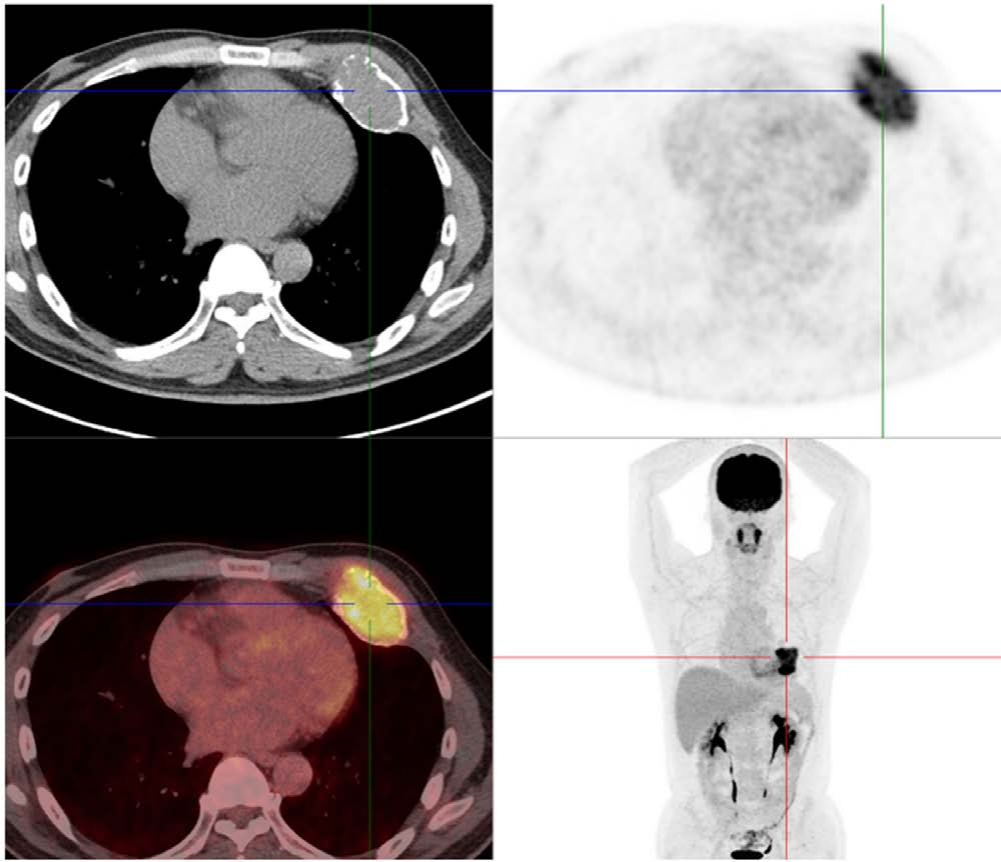
Fluorine-18-FDG PET showed that the size of the mass was about 11 × 11 × 9 cm, several high-density shadows similar to calcified lesions, uneven density, unclear outline of intercostal muscles, widened intercostal space of the fifth and sixth ribs, and the CT value was between 15 and 50. CT = computed tomography, FDG = fluorodeoxyglucose, PET = positron emission tomography.

**Figure 2.**
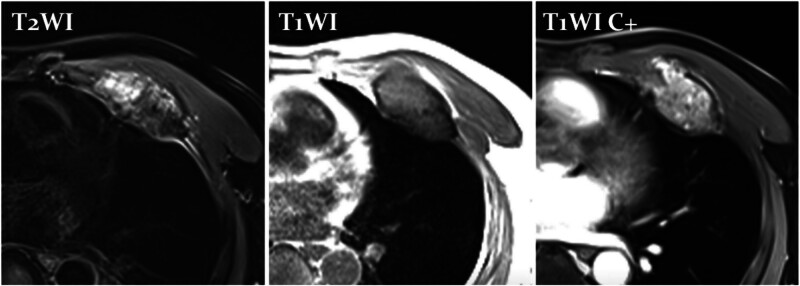
Magnetic resonance imaging showed conventional chondrosarcoma on MRI showed medium-low signal on T1WI and uneven high signal on T2WI. MRI = magnetic resonance imaging.

Physical examination showed that a hard mass with approximately a size of 13 × 10 cm could be touched at the intersection of the 5th to 7th ribs of the left chest wall and the left midaxillary line, with unclearly border and unable movement. There is no redness or swelling of the local skin and no obvious varicose veins. He underwent a chest CT examination in the external department. The CT scan revealed a low-density mass measuring 10.6 × 9.8 × 8.9 cm on the left chest wall. It was lobulated in shape, with bone-like calcifications at the boundary, and the contour remained clear. There were sand-like calcifications within the lesion. Fluorine-18-fluorodeoxyglucose (FDG) positron emission tomography showed that the mass size was about 11 × 11 × 9 cm, partial damaged in the 6th rib bone cortex such as thickening and roughening, the CT value of the mass arranged from 15 to 50, and the SUV max was 2.8 (Fig. 1). MRI showed Conventional chondrosarcoma on MRI showed medium-low signal on T1WI and uneven high signal on T2WI (Fig. 2). Based on his medical history and imaging findings, the radiology diagnostician almost diagnosed him as having a benign bone tumor, specifically an osteochondroma.

**Figure 3.**
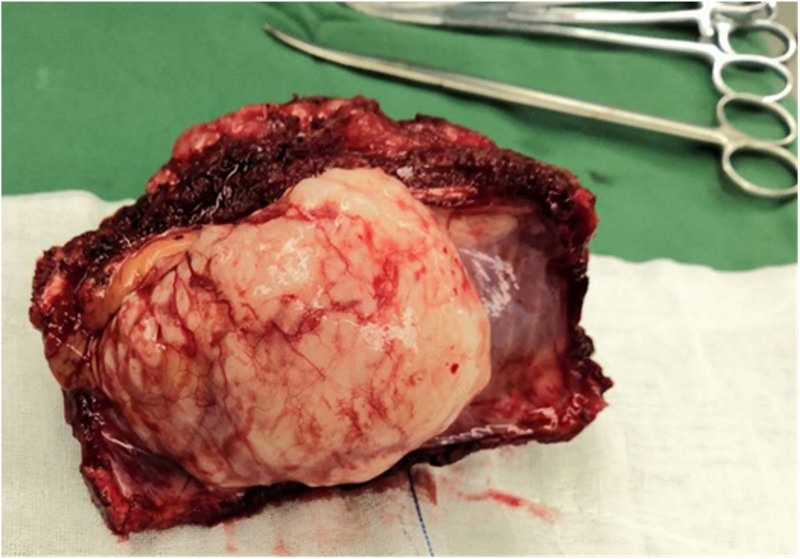
The tumor protruding in the thoracic cavity was semicircular, with a grayish white appearance, and the surface was covered by a smooth envelope. The rich blood vessels on the tumor surface could be vaguely seen.

**Figure 4.**
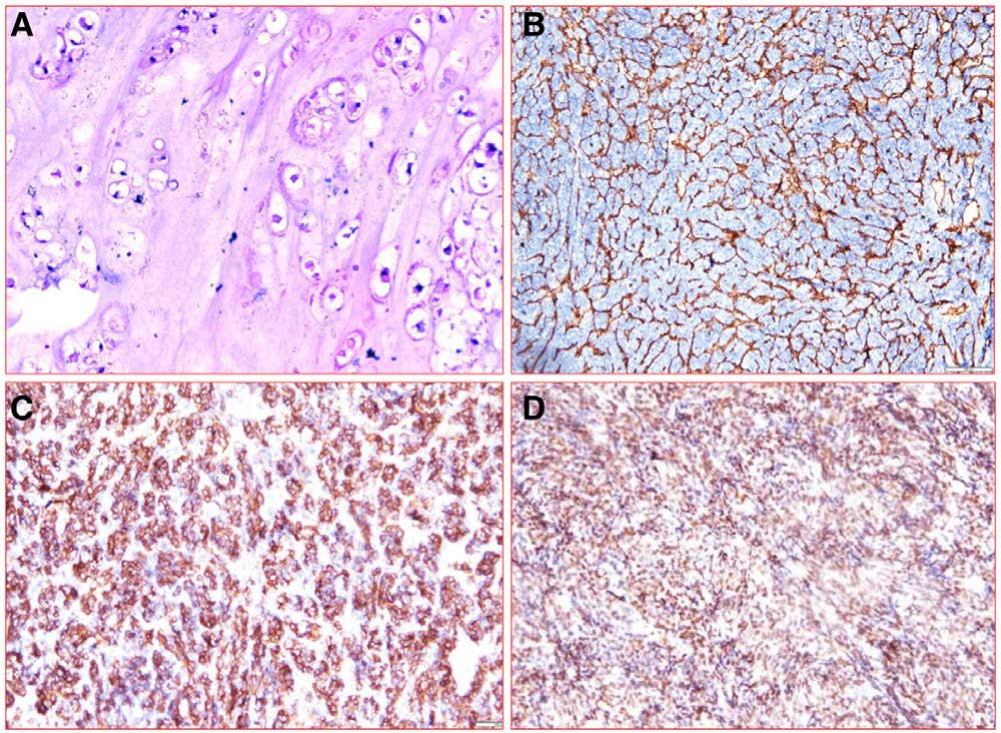
Histological sections showed a large number of clear chondroid stroma and atypical chondrocytes, with a small number of mitotic cells. (A) hematoxylin-eosin stained, original × 200; (B) CD34 × 100; (C) CD99 × 40; (D) TLE1 × 100.

Based on this, the multidisciplinary team (MDT) discussion group recommended surgical treatment. The plan is to perform resection of the tumor and reconstruction of the chest wall for the patient. The operation was performed under general anesthesia and the patient was placed in the lateral position. He was made an incision at the sixth rib of the left midaxillary line. During the operation, it was found that the tumor covered by 1-layer envelope and did not invade the subcutaneous tissues and muscles, but about 10 cm length of 5th and 6th ribs were surrounded by the tumor and the tumor broke through the visceral pleura and grew into the pleural cavity. Fortunately, the left lung was not invaded (Fig. 3). We completely resected the tumor along the boundary 1 cm outside the tumor invasion margin. The resected lengths of the 5th and 6th ribs were both approximately 15 cm. After sufficient hemostasis, the defective chest wall was reconstructed with polyester patches and poly (p-dioxyhexanone) sutures. Two drainage tubes were retained before the incision was closed. One of them is located in the thoracic cavity, and the outer end is connected to a closed drainage bottle. The other 1 is located in the surgical area of the chest wall, with a negative pressure drainage ball attached to the outer end. The intraoperative rapid freezing pathology results indicated that the type of the tumor could not be confirmed (frozen sections could not be performed because the tumor contains hard bone tissue), but all the edges of the cut off the tumor were negative. Postoperative histopathological report indicated grade 1 chondrosarcoma (Fig. 4). Three days after the operation, the drainage volume of the patient was low and the drainage tube was removed. Ten days after the operation, the patient recovered well and discharged from the hospital without collapse of the left chest wall. Considering the slow growth of the tumor, this patient did not receive any other adjuvant treatments after the surgery, including chemotherapy or targeted therapy. After 5 years of follow-up, several chest CT reexaminations were conducted on the patient, and no tumor recurrence or metastasis was found. He was pleased with the result of the treatment.

## 3. Discussion

Chondrosarcoma was a rare type of malignant tumor with an incidence rate of 2 parts per million.^[4]^ Generally, the incidence of the disease was higher in people aged 30 to 60 years, and there was no difference between men and women.^[4]^ The affected sites of chondrosarcoma general were pelvis, femur, humerus and ribs in that order.^[7]^ The causative factors and pathogenesis of chondrosarcoma are still unclear. At present, there are no specific imaging and serological diagnostic indicators, and it is mainly diagnosed by histopathology. From the perspective of histology, chondrosarcoma could be divided into grade 1, grade 2, and grade 3,^[8]^ and the corresponding 10-year survival rates were 79% to 100%, 53% to 90%, and 29% to 55%.^[9]^ In order to underline the characteristics of low-grade malignant chondrosarcoma, the World Health Organization in its fifth edition of classification of tumors of soft tissue and bone on the 2020 identified grade 1 chondrosarcoma as an atypical cartilage tumor.^[10]^

Rib chondrosarcoma develops rapidly and mostly occurred in middle-aged or elderly crowds. Rib chondrosarcoma in children is a rare. In this case, the mass has existed since birth and has grown symptomatically for 30 years without metastasis, which is extremely rare. Such a slow growth pattern of rib chondrosarcoma has not been reported in the previous literature. Some reported chondrosarcomas were generally asymptomatic at first, but most patients came to medical institutions because of the gradual enlargement of a painless mass.^[11]^ In this case, the pain and numbness of the chest wall probable related to the invasion of the chest wall or the compression of subcutaneous nerves which caused by excessive tumor growth. The slow growth pattern of rib chondrosarcoma is a critical aspect that influences both diagnosis and treatment outcomes. Chondrosarcoma, particularly in the rib area, could be insidious growth, which can complicate early detection. The slow growth nature of these tumors means that they can reach significant sizes before symptoms arise, leading to challenges in management. Moreover, the histological characteristics of rib chondrosarcoma can vary, with some cases exhibiting a slow-growing pattern that mimics benign conditions. This was observed in a case where a dedifferentiated chondrosarcoma resembled a giant cell tumor, underscoring the importance of accurate histological evaluation to determine the appropriate treatment strategy.^[12]^ The slow growth can also impact the likelihood of local recurrence and metastasis, as seen in conventional chondrosarcoma, where slow-growing tumors may still pose significant risks if not adequately treated.^[13]^ In addition, the microenvironment and biological behavior of rib chondrosarcoma contribute to its slow growth pattern. Research indicates that the extracellular matrix and signaling pathways play crucial roles in the growth dynamics of chondrosarcomas, which can affect how these tumors respond to treatment.^[14]^ Understanding these factors is essential for developing effective therapeutic approaches and improving patient outcomes.

Due to the small amount of information provided and lack of specificity, chest X-ray examinations are currently mainly used for general physical examination screening in most medical institutions to provide a basis for further CT and MRI examinations. Primary grade 1 chondrosarcoma of ribs presented an uneven density mass on chest CT, calcified focal spot might occur or not occur in the mass, and bone destruction might be observed.^[15]^ Researches had shown that the presence of calcifications in the mass was a vital predictor of good prognosis.^[16]^ On MRI, the chondrosarcoma was generally manifested as soft tissue edema, cortical bone thickening, and bone destruction. In addition, some studies believed that MRI could be used to distinguish the histological grade of chondrosarcoma of the long bone.^[17]^ In addition, preoperative enhanced CT and 3D reconstruction could better display the blood supply and the 3D image of the tumor in the chest wall, which could better guide the surgeon to perform surgical excision.

Surgical resection was essential to the management of grade 1 chondrosarcoma, due to this type chondrosarcoma was not sensitive to chemotherapy and radiation.^[5]^ Therefore, negative result of incisal margins around the tumor were the top priority.^[18]^ A single-center study had shown strong correlation between incisal margins and postoperative prognosis.^[11]^ At present, there is some controversy in the opinion of taking local resection or expanded resection in grade 1 chondrosarcoma. Therefore, researches had shown that local resection and expanded resection had no statistical significance in postoperative recurrence and prognosis, while local resection could reduce postoperative complications in patients.^[19]^ Reconstruction of the chest wall following resection is a critical component of the surgical procedure, as it ensures structural stability and protects vital organs. Various materials and techniques can be employed for reconstruction, including the use of titanium mesh, which provides essential rigidity and minimal elasticity, as demonstrated in a case involving the anterior chest wall.^[20,21]^ Alternatively, polypropylene mesh has been used successfully in pediatric cases, offering a flexible yet durable solution for chest wall reconstruction.^[22]^

There have been some recent advances in research suggest that isocitrate dehydrogenase 1 gene and isocitrate dehydrogenase 1 gene mutations have been detected in most cases of grade 1 chondrosarcoma, which provided more means for the treatment of the disease.^[23]^ So, recent advancements in systemic therapies and targeted treatments are opening new avenues for improving outcomes in patients with this disease. Isocitrate dehydrogenase inhibitors have shown promising efficacy in preclinical and early clinical trials, although data specific to chondrosarcoma remains limited.^[24]^ Additionally, the hedgehog signaling pathway, which plays a role in chondrosarcoma progression, is being targeted with inhibitors, though clinical results have been mixed.^[24]^ Immunotherapy is another emerging field in the treatment of advanced chondrosarcoma. Despite the challenges posed by the immunosuppressive tumor microenvironment, therapies such as programmed cell death 1 (PD-1) checkpoint inhibitors and chimeric antigen receptor-T (CAR-T) cells are under investigation. These approaches aim to enhance the body’s immune response against the tumor cells.^[24]^

Chondrosarcoma, particularly when located in the rib, presents significant challenges in treatment due to its resistance to conventional therapies like chemotherapy and radiotherapy.^[25]^ The role of chemoradiation in managing rib chondrosarcoma is an area of ongoing research, as these tumors are typically resistant to standard radiation doses. However, recent studies have explored various strategies to enhance the effectiveness of radiotherapy, including the use of carbon ion radiotherapy and radiosensitizers. Carbon ion radiotherapy has been shown to have a precise dose distribution and high biological effectiveness, making it a promising option for treating unresectable chondrosarcomas. In addition to advanced radiotherapy techniques, the combination of radiotherapy with radiosensitizers has been investigated. For instance, disulfiram, an FDA-approved anti-alcoholism drug, complexed with copper, has been shown to radiosensitize cancer stem cells in chondrosarcoma, potentially overcoming the radioresistance observed in these tumors. This combination was found to be more effective than radiotherapy alone in inhibiting the growth of chondrosarcoma xenografts, indicating that such approaches could enhance the efficacy of radiotherapy in rib chondrosarcoma.^[26]^ Moreover, multimodal treatment strategies that combine carbon ion irradiation with molecular treatments, such as miRNA-34 and mTOR inhibitors, have been explored to target cancer stem cells in high-grade chondrosarcoma. These approaches have shown promise in overcoming treatment resistance and controlling cancer stem cells, which are often responsible for relapse and metastasis.^[27]^ Such strategies highlight the potential of integrating chemoradiation with targeted therapies to improve outcomes in rib chondrosarcoma.

Overall, the integration of new systemic therapies, targeted biological treatments, and advanced surgical techniques holds promise for improving the prognosis of patients with rib chondrosarcoma. Surveillance and follow-up are crucial after surgical treatment of rib chondrosarcoma to monitor for recurrence and manage any complications. Regular imaging, such as CT scans, is often employed to assess the surgical site and detect any signs of tumor recurrence early.

## 4. Conclusions

Overall, the slow growth pattern of rib chondrosarcoma is a multifaceted issue that requires careful consideration in clinical practice. The clinical symptoms are mainly manifested as a gradually enlarged hard chest wall mass, which may be accompanied by other symptoms, such as local pain. Based on these clinical symptoms and biological characteristic, clinicians are susceptible to misdiagnosing this disease. Early diagnosis, accurate histological assessment, and an understanding of the tumor’s biological behavior are vital for effective management and treatment planning.

## Acknowledgments

The authors thank the patient who agreed to be included in this study.

## Author contributions

**Data curation:** Mei Shen, Ying Liu, Wei Wen, Hanlong Guo, Qu-Cheng Huang.

**Formal analysis:** Mei Shen, Wei Wen.

**Funding acquisition:** Wei Wen, Wen-Juan Liao.

**Investigation:** Wei Wen, Wen-Juan Liao.

**Methodology:** Mei Shen, Ying Liu, Hanlong Guo, Qu-Cheng Huang.

**Project administration:** Ying Liu, Wei Wen, Wen-Juan Liao.

**Software:** Ying Liu.

**Supervision:** Ying Liu, Wei Wen.

**Validation:** Mei Shen, Ying Liu.

**Visualization:** Hanlong Guo.

**Writing – original draft:** Mei Shen, Ying Liu.

**Writing – review & editing:** Wei Wen, Wen-Juan Liao.
